# Transcriptomic Insight in the Control of Legume Root Secondary Infection by the *Sinorhizobium meliloti* Transcriptional Regulator Clr

**DOI:** 10.3389/fmicb.2017.01236

**Published:** 2017-07-06

**Authors:** Lan Zou, Amandine Gastebois, Céline Mathieu-Demazière, Fernando Sorroche, Catherine Masson-Boivin, Jacques Batut, Anne-Marie Garnerone

**Affiliations:** LIPM, Université de Toulouse, INRA, CNRSCastanet-Tolosan, France

**Keywords:** rhizobium, legume, symbiosis, infection, adenylate cyclase, cAMP, transcriptome

## Abstract

The cAMP-dependent transcriptional regulator Clr of *Sinorhizobium meliloti* regulates the overall number of infection events on *Medicago* roots by a so-far unknown mechanism requiring *smc02178*, a Clr-target gene of unknown function. In order to shed light on the mode of action of Clr on infection and potentially reveal additional biological functions for Clr, we inventoried genomic Clr target genes by transcriptome profiling. We have found that Clr positively controls the synthesis of cAMP-dependent succinoglycan as well as the expression of genes involved in the synthesis of a so-far unknown polysaccharide compound. In addition, Clr activated expression of 24 genes of unknown function in addition to *smc02178*. Genes negatively controlled by Clr were mainly involved in swimming motility and chemotaxis. Functional characterization of two novel Clr-activated genes of unknown function, *smb20495* and *smc02177*, showed that their expression was activated by the same plant signal as *smc02178 ex planta*. *In planta*, however, symbiotic expression of *smc02177* proved independent of clr. Both *smc02177* and *smb20495* genes were strictly required for the control of secondary infection on *M. sativa*. None of the three *smc02177, smc02178* and *smb20495* genes were needed for plant signal perception. Altogether this work provides a refined view of the cAMP-dependent Clr regulon of *S. meliloti*. We specifically discuss the possible roles of *smc02177, smc02178, smb20495* genes and other Clr-controlled genes in the control of secondary infection of *Medicago* roots.

## Introduction

*Sinorhizobium meliloti* is a gram-negative bacterium that alternates between a free-living saprophytic life in the soil and the rhizosphere of plants, and an occasional symbiotic life within nodules of *Medicago* spp. Establishment of symbiosis requires the coordinated bacterial infection of the root epidermis and initiation of nodule organogenesis in the root cortex (Oldroyd, 2013). Neo-formed nodules are invaded intracellularly by bacteria that differentiate into nitrogen-fixing bacteroides. Infection of root hair cells takes place via specialized structures called epidermal Infection Threads (eIT) (Miri et al., 2016). Two bacterial effector molecules are required for eIT formation: Lipo-chitooligosaccharides (LCOs, also known as Nod factors), which are also needed for nodule organogenesis, and exopolysaccharides such as succinoglycan in *S. meliloti* (Cheng and Walker, 1998; Jones et al., 2007; Gibson et al., 2008).

Mechanisms negatively controlling the symbiotic interaction have also been identified. The best known is AON (autoregulation of nodulation) by which the plant systemically adjusts the number of nodules to its metabolic needs (Magori and Kawaguchi, 2009; Mortier et al., 2012). We recently obtained preliminary evidence that eIT formation was also negatively autoregulated in the *S. meliloti–Medicago* symbiosis (Tian et al., 2012). Specifically, we isolated *S. meliloti* mutants that displayed a hyper-infection phenotype on *Medicago* roots, resulting from a relaxed control of eIT formation. Whereas eIT formation by wild-type bacteria is transient, mutants kept infecting roots for an extended time-lapse, thus allowing increased secondary infection. Noteworthy, nodulation by hyper-infecting bacterial mutants was normal thus indicating that autoregulation of infection and nodulation are distinct mechanisms.

In *S. meliloti*, the control of secondary infection is mediated by a bacterial cAMP-cascade involving three receptor-like adenylate cyclases (ACs), CyaD1 (SMc02176), CyaD2 (SMc04307) and CyaK (SMb20776), a cAMP-dependent transcriptional regulator of the Crp family (Green et al., 2014), called Clr (SMc02175), and *smc02178*, a gene of unknown biochemical function located nearby *cyaD1* and *clr* on the chromosome. In nodules, a so-far unknown plant signal activates cAMP production by CyaD1, CyaD2 and CyaK, which in turns allows activation of *smc02178* transcription by Clr. This signal is also present in shoots but very low in roots (Tian et al., 2012). We and others have shown that purified Clr is as a 3′5′cAMP-dependent DNA-binding protein that binds a Clr-box in the promoter region of the *smc02178* gene (Mathieu-Demaziere et al., 2013; Krol et al., 2016). Inactivation of *clr*, of *smc02178* or of the three AC genes altogether, resulted in a hyper-infection phenotype on *M. sativa* (Tian et al., 2012). Noteworthy, individual mutants of any of the three ACs had no conspicuous hyper-infection phenotype thus indicating all three genes contribute full infection control, probably upon acting at different stages of the symbiotic interaction (Tian et al., 2012).

In order to shed light on the mechanism by which *S*. *meliloti* bacteria control secondary infection, we have identified here additional Clr targets by transcriptome profiling. We have found that Clr positively controls expression of 25 genes of unknown function as well the synthesis of succinoglycan and of a putative unknown polysaccharide. We compare our results with those recently obtained upon over-expression of CyaJ (Krol et al., 2016). Functional characterization of two genes of unknown function, *smc02177* and *smb20495*, demonstrated their implication in the control of secondary infection. We discuss the possible roles of *smc02177, smc02178, smb20495* and Clr-controlled surface polysaccharides in the control of secondary infection of *Medicago* roots.

## Materials and Methods

### Bacterial Strains and Culture Conditions

Bacterial strains used in this study are listed in Supplementary Table 1. Unless otherwise indicated, strains were grown at 28°C in VMG medium, i.e., Vincent minimal medium (Becker et al., 2004), supplemented with mannitol (1%w/vol) and glutamate (0.1%) as carbon and nitrogen sources, respectively. The concentrations of antibiotics used for *S. meliloti* were 200 μg/ml for streptomycin, 100 μg/ml for neomycin and 10 μg/ml for tetracycline in both liquid and solid media. Gentamicin was used at 10 μg/ml in liquid medium and 30 μg/ml on solid medium.

Primers used for DNA amplification are listed in Supplementary Table S2. *S. meliloti* Rm1021 was used as template for DNA amplification. The *smb20495* and *smc02177* single mutants were constructed by site-specific integration of the suicide pVO155 plasmid. *smb20495* and *smc02177* internal PCR fragments were amplified using 20495L-20495R, and L02177-R02177 primers (Supplementary Table S2), cloned into pCR2-1-TOPO and digested with *Bam*HI and *Xba*I before cloning into pVO155. The resulting pVO155 derivatives were introduced into *E. coli* DH5α by transformation and then conjugated in *S. meliloti* using pRK600 as helper plasmid. All constructs were verified by PCR and Sanger sequencing in *E. coli* and by PCR in *S. meliloti*.

### Plasmids Construction

Plasmids used in this study are listed in Supplementary Table S1. The *clr*-overexpressing construct pFA2175 was obtained after PCR amplification of the *clr* gene coding region using *S. meliloti* Rm1021 genomic DNA as template and the REco2175 and LBamH2175 primers (Supplementary Table S2). The PCR fragment was digested with BamHI and EcoRI and ligated into a BamHI-EcoRI digested pFAJ1708 plasmid. For pGD926-ExoY construction, the promoter region and the first 297nt of *exoY* coding region were PCR-amplified from pstb-LAFR5-ExoY plasmid DNA using the ExoY-fusHindIII and ExoY-fusBamHI primers. The PCR fragment was digested with BamHI and HindIII and ligated into a BamHI-HindIII digested pGD926 plasmid.

For Lsmc02178-lacZ construction (pGMI50322), the *smc02178* coding region including signal peptide was amplified using the 2178H and 2178BamHIlacZ primers. The HindIII-BamHI resulting PCR product was subsequently cloned into HindIII and BamHI-digested pGD926. For the *phoA* fusions, the signal-peptide containing fusion (Lsmc02178-phoA) was obtained using the primers 2178H and 2178EcoRVphoAL whereas the short construction (Ssmc02178-phoA) lacking endogenous signal peptide was obtained using the 2178H and 2178EcoRVphoAc primers. In parallel, the *phoA* gene from *E. coli* genomic DNA was amplified using the primers phoAEcoRV and phoABamHI and cloned into pGEM plasmid. The *phoA* and Lsmc02178 or Ssmc02178 fragments were ligated together, PCR-amplified, digested by Hind III and BamHI before cloning into HindIII/BamHI digested-pGD926. Constructs were conjugated into a *phoA S. meliloti* derivative (Rm8002) to minimize phosphatase alkaline background activity. For the construction of pGD20495, a 934 bp PCR fragment encompassing the *smb20495* promoter region (full intergenic region) and 26 nucleotides of the *smb20495* gene was amplified using the SMb20495-HindIII and SMb20495-BamHI primers, digested with BamHI and HindIII and cloned in-frame with *lacZ* in the translational fusion plasmid pGD926 For pGD2177, a 303 bp PCR fragment encompassing the full intergenic region between *smc02177* and *smc02178* and 32 nucleotides of the *smc02177* gene was amplified using the p2177HindIII and p2177BamHI primers, digested with BamHI and HindIII and cloned in the in-frame orientation at the same sites of the *lacZ* translational fusion plasmid pGD926.

### Transcriptome and Quantitative PCR Analyses

For transcriptome experiments, *S. meliloti clr*^-^ (GMI11567) and *clr*-overexpressing (GMI11896) strains (**Table 1**) were grown overnight at 28°C in VMG medium, diluted to an OD600 of 0.12 in 20 ml of VMG medium and incubated under either oxic or microoxic (2%O_2_) conditions in the presence of caffeine (2.5 mM), to inhibit endogenous phosphodiesterase activity (Essayan, 2001). Microoxic conditions were achieved as described before (David et al., 1988). When OD600 reached 0.3, 15 ml were filtered on Supor^®^ Membrane Disk Filters (Pall), and stored at -80°C. RNA preparations were as described in Bobik et al. (2006).

**Table 1:** Clr-induced and Clr-repressed genes.

Gene ID	Gene product	*M*-value Clr++/clr- Aerobic	*M*-value Clr++/clr- Microaerobic
**Induced genes**			
**smb20495^∗^**	Conserved hypothetical protein	4,585	5,5721
**smc04190^∗^**	Conserved hypothetical protein	4,0758	4,8696
smb21224^∗^	NodQ2 sulfate adenylyltransferase protein	2,0767	3,8393
smc01589	Hypothetical protein	2,1773	3,7637
smc02278^∗^	Hypothetical transmembrane protein	4,0335	3,523
sma0855^∗^	NodP1 ATP sulfurylase	1,453	3,5207
smb21671^∗^	Hypothetical protein	2,2859	3,3252
**smc03985**	CyaF2 adenylate/guanylate cyclase protein	2,4864	3,1647
smb21223^∗^	NodP2 sulfate adenylyltransferase protein	1,3894	3,116
**smc02178^∗^**	Hypothetical protein	2,1725	2,9945
*smc02175*	*Clr, transcription regulator protein*	*2,4063*	*2,95*
**smb20907^∗^**	Hypothetical protein	2,1938	2,688
smc00198^∗^	Hypothetical protein	2,0852	2,5524
**smc04164^∗^**	Hypothetical protein	1,4981	2,466
**sm00864^∗^**	Hypothetical protein	1,556	2,463
smc03100^∗^	Hypothetical protein	2,7891	2,4427
**smc01210^∗^**	Hypothetical protein	1,2358	2,3854
smb20960^∗^	ExoN UDP glucose pyrophosphorylase protein	1,1766	2,3588
**smc02177^∗^**	Hypothetical protein	1,5143	2,3533
smb21240^∗^	Putative surface saccharide export protein	1,5351	2,2783
smb21242^∗^	Putative glycosyltransferase protein	1,6205	2,2734
smb20409	Putative hydroxybutyrate dehydrogenase	1,9187	2,2027
smc01764	Hypothetical protein	1,383	2,1944
smb21243	Putative sulfotransferase protein	1,0488	2,14
smc03810	Conserved hypothetical protein	1,8922	2,1328
smb21248	Putative aminotransferase protein	1,5844	2,1101
**smb21329**	Hypothetical exported peptide protein	1,8956	2,066
smb21069^∗^	Hypothetical beta-glucosidase protein	1,3609	2,0346
smb21247^∗^	Conserved hypothetical protein	1,7569	2,0059
smc01003^∗^	Hypothetical protein	1,0864	1,9794
smb20954	ExoH succinyltransferase protein	1,093	1,8886
smc00639	Putative heat resistant agglutinin 1 protein	1,2183	1,8819
smb20946^∗^	ExoY galactosyltransferase protein	1,1735	1,8564
smc01855	Hypothetical transmembrane protein	1,7977	1,7555
smc04267	LpsS LPS sulfotransferase	1,0996	1,6751
smb20945^∗^	ExoF1 polysaccharide export protein	1,1785	1,6738
**smc01136^∗^**	Hypothetical transmembrane protein	1,0215	1,6632
smb21690^∗^	ExoW glucosyltransferase protein	1,2603	1,4455
smc01241	Hypothetical protein	1,3629	1,3209
smb20948	ExoU glucosyltransferase protein	1,1547	1,3011
**smb20908**	Hypothetical protein	1,1748	1,2806
smc01580^∗^	Hypothetical transmembrane protein	1,0209	1,0248
**smc00925^∗^**	Hypothetical protein	1,378	0,9256
**Repressed genes**			
smc03027^∗^	FlgB flagellar basal body rod protein	-2,2764	-2,9643
smb20745	GlnII putative glutamine synthetase II protein	-2,4136	-2,7335
smc03009	CheR chemotaxis protein methyltransferase	-1,4759	-2,4545
smc03049^∗^	FlgL putative flagellar hook associated protein	-1,08	-2,1779
smc02251	Putative aminotransferase protein	-1,3324	-1,9797
smc04114^∗^	PilA1 putative pilin subunit protein	-1,3701	-1,9591
smc01122	Conserved hypothetical protein	-1,2814	-1,7944
smb20025	Conserved hypothetical protein	-1,7088	-1,716
smc01468	CheW2 chemotaxis protein	-1,717	-1,6945
smb20293	Hypothetical protein	-1,3861	-1,645
smc01104	McpX methyl accepting chemotaxis protein	-1,8696	-1,6241
smc03121	Putative periplasmic ABC transport protein	-1,4263	-1,5996
smc01795	Polysaccharide synthesis/transport protein	-1,2805	-1,5858
smc00987	Conserved hypothetical protein	-1,8713	-1,5637
smc03017	Conserved hypothetical protein	-1,6966	-1,5628
sma1507	Conserved hypothetical protein	-1,3878	-1,5539
smc03008	CheW1 chemotaxis protein	-1,1378	-1,4239
smc02146	Putative phosphate binding periplasmic protein	-1,4216	-1,382
smc00787	DppB1 dipeptide transport permease protein	-1,1378	-1,3779
smc03806	GlnK nitrogen regulatory protein PII	-1,3264	-1,3172
smc03072	Conserved hypothetical protein	-1,5406	-1,2392
smc01848	Conserved hypothetical protein	-1,4597	-1,2183
smc03052	FlgD basal body rod modification protein	-1,1339	-1,2173
smb20263	Putative ABC transporter periplasmic protein	-1,177	-1,1958
smc03046^∗^	Putative transcription regulator protein	-1,0913	-1,149
smc02588	Putative permease ABC transport protein	-1,0468	-1,1399
smc01957	Conserved hypothetical protein	-1,0356	-1,0588
smc00330	Putative transmembrane protein	-1,312	-1,0338
smc01949	LivG branched aminoacid transport protein	-1,0369	-1,0298
smc01931	Conserved hypothetical transmembrane protein	-1,0284	-1,0104

*Genes showing up- or down-regulation in both aerobic or microoxic conditions in two biologically independent replicates are listed. ^∗^Indicates the genes common to Krol et al. (2016). Genes displaying a Clr-box in their promoter are in bold face (Supplementary Table S3). Raw data are available in the ArrayExpress database (www.ebi.ac.uk/arrayexpress) under accession number E-MTAB-4780.*

Transcriptome analyses were performed using 70-mer oligonucleotide microarrays representative of 6,207 predicted open reading frames (ORFs) of *S. meliloti* Rm1021. The protocol was as described before (Bobik et al., 2006). Microarray raw data have been deposited in the ArrayExpress database under accession number E-MTAB-4780.

**FIGURE 1:**
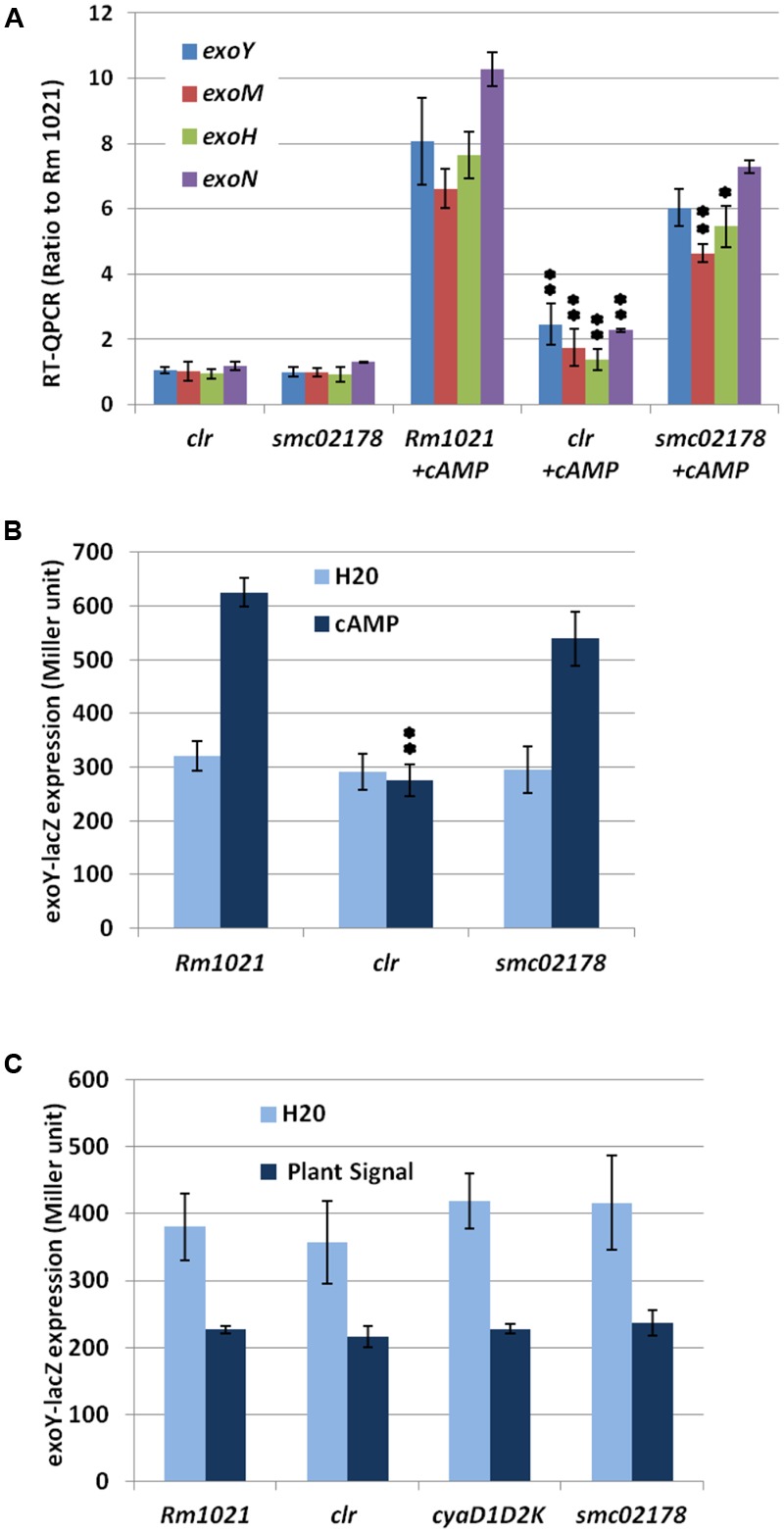
Clr and cAMP control succinoglycan production. **(A)** RT-qPCR analysis of *exo* genes expression in response to 5 mM _exo_cAMP, expressed as ratio to Rm1021 wt strain. **(B)** Expression of a translational *exoY-lacZ* reporter fusion in the presence of 10 mM _exo_cAMP. **(C)** Expression of a translational *exoY-lacZ* reporter fusion in the presence of plant shoot extract. *smc02178-lacZ* expression was included as an internal control for plant shoot extract activity but data were omitted in **(C)** for clarity. ^∗^ and ^∗∗^ indicate statistical significance at *p* ≤ 0.05 and *p* < 0.01, respectively, with respect to Rm1021+cAMP.

For _exo_cAMP-induction assays of *exo* and *flaB* gene expression (**Figure 1** and Supplementary Figure S2, respectively), overnight cultures of *S. meliloti* strains at 28°C in VMG medium were diluted to an OD600 of 0.12 in 20 ml of same medium and supplemented with 5 mM (final concentration) of 3′-5′-cAMP (Sigma) when cultures reached an OD600 of 0.3. When _exo_cAMP-treated cultures reached an OD600 of 0.6, 15 ml were filtered on Supor^®^ Membrane Disk Filters (Pall) and cells were frozen in liquid nitrogen and stored at -80°C. All experiments were performed in triplicate.

Reverse transcriptions were performed from 1 μg of RNA using the Transcriptor Reverse Transcriptase (Roche), and random hexamers as primers. cDNAs were used for running real-time PCR on a LightCycler 1.5 system (Roche) using the FastStart DNA Master PLUS SYBRGreen I kit (Roche) according to the manufacturer’s instructions. The *rplM* gene was used as reference gene for data normalization. Gene expression was assessed using the second derivative maximum analysis.

### β-Galactosidase and Alkaline Phosphatase Assays

*Sinorhizobium meliloti* strains carrying the pGD926-ExoY, pGD2178, pGD20495 or pGD2177 plasmids were grown at 28°C in VMG medium. Overnight cultures were diluted to an OD600 of 0.1 in 10 ml Vincent medium and additionally grown for 2 h. Cultures were then supplemented with 10 mM _exo_cAMP for pGD926-ExoY (**Figure 1B**) and grown for an additional 24 h. *S. meliloti* strains carrying plasmids pGMI50322, pGMI50323, pGMI50324, and pGD2178 were grown overnight in 1 ml-cultures in VMG medium supplemented with 100 μl of *Medicago* shoot signal fresh extract prepared as described in Tian et al. (2012). Although a plant shoot extract is slightly less active than a nodule extract (Tian et al., 2012) it was preferred as it is easier to prepare ex temporaneously.

The assays for beta-galactosidase activity were carried out using the protocol of Miller (1972), whereas alkaline phosphatase was assayed in a *S. meliloti phoA* background as described before (Brickman and Beckwith, 1975). All experiments were performed in triplicate.

Symbiotic detection of beta-galactosidase activity in *Medicago* nodules was conducted as described before (Tian et al., 2012).

### Calcofluor Dye Detection of Succinoglycan

*Sinorhizobium meliloti* strains were grown at 28°C in LB-MC (LB+2.5 mM MgCl_2_ and 2.5 mM CaCl_2_). Overnight cultures were diluted to an OD600 of 0.15 and 10 μl were dropped off as a spot on LB-MC plates supplemented with 0.02% calcofluor white (Fluorescent Brightener 28, Sigma). After 2 and 13 days incubation, the fluorescence was monitored under UV light with a G:BOX BioImaging System (Syngene).

### Motility Assays

10^4^ cells (*ca* 5 μl) from overnight cultures in LB-MC medium were jabbed on top of soft (0.2%) agar plates. Motility (i.e., size of the colony) was measured after 5 days at 28°C.

## Results

### Transcriptome Profiling of the Clr Regulon

We compared the transcriptomes of a *S. meliloti* strain (GMI11896) expressing *clr* from a constitutive *nptII* promoter on a low copy-number plasmid and of a nearly isogenic *clr* null mutant strain (GMI11567). Experiments were performed under both free-living aerobic and microoxic conditions, as low oxygen is a known symbiotic signal (Soupene et al., 1995). Both experiments, performed in biological duplicate, revealed a similar list of genes with the same extent of induction or repression thus indicating that oxygen did not affect Clr activity. We identified 72 genes displaying at least a twofold change in gene expression in both oxic and microoxic conditions, i.e., in 4 independent assays (**Table 1**). Most of these genes are probably indirect Clr targets (see below).

*Sinorhizobium meliloti* has a tripartite genome consisting of one main chromosome (3.65 Mb) and two symbiotic replicons called pSymB (1.68 Mb) and pSymA (1.35 Mb) (Galibert et al., 2001). The 42 genes activated by Clr were all located on pSymB and chromosome with the exception of the pSymA-located *nodP1* gene which was likely a false positive that was detected because of its very strong (99.7%) sequence similarity with *nodP2* (see below).

Targets included six genes involved in succinoglycan synthesis (*exoN, Y, H, F1, W, U*), an exopolysaccharide that plays a key role in IT formation and stress adaptation.

Seven genes belong to a large *ca*. 30-kb cluster on pSymB between genes *nodP2* and *smb21248* that contains many genes involved in polysaccharide metabolism (see *S. meliloti* genome browser^1^), (Galibert et al., 2001).

Clr-targets in this cluster encompass two categories of genes: three genes involved in activated sulfate (PAPS) biosynthesis and sulfate transfer (NodP2, NodQ2, SMb21243) and four genes involved in sugar/polysaccharide metabolism including a glycosyl transferase (SMb21242), a beta-glucosidase (SMb21069), a sugar (GlcNAc-containing) deacetylase (SMb21247) and Smb21240, a protein with a tyrosine kinase domain resembling surface exopolysaccharide export protein of the ExoP family. Altogether, Clr target genes in this cluster may contribute synthesis of a sulfated surface polysaccharide (see Discussion).

Additional Clr targets included the *lpsS-*encoded sulfotransferase (Cronan and Keating, 2004), *cyaF2* encoding an adenylate/guanylate cyclase of unknown function, as well as 25 genes of unknown function (including *smc02178*), most of which were located on the main chromosome.

Thirty genes were repressed by Clr under both oxic and microoxic conditions, most of which (25) were chromosomal (**Table 1**). Ten genes belonged to a large chemotaxis/motility cluster (from *smc03004* to *smc03072*) involved in flagella synthesis, swimming motility and chemotaxis. Related genes were *smc01104* (*mcpX*), *smc01468* (*cheW2*) and *smc04114* (*pilA1*). We also detected two genes involved in nitrogen metabolism (*glnK, glnII*) and 11 genes of unknown function. We identified a conspicuous Clr-box in the promoter region of 12 Clr target genes (**Table 1** and Supplementary Table S3, see Discussion).

### Clr and cAMP Drive Succinoglycan Synthesis

RT-qPCR analysis of four *exo* genes (*exoH, exoM, exoN*, and *exoY*) in *S. meliloti* wt and *clr* mutant cells supplemented with exogenous cAMP (abbreviated thereafter as _exo_cAMP) (**Figure 1A**) confirmed that the *clr* gene indeed mediated _exo_cAMP-activation of these genes. Inactivation of the direct Clr-target *smc02178* had no significant effect on *exoY* and *exoN* expression but slightly decreased *exoM and exoH* gene expression (**Figure 1A**). Expression of a translational *exoY-lacZ* reporter fusion (**Figure 1B**) confirmed the *clr*-dependency and *smc02178*-independency of the _exo_cAMP-mediated *exoY-lacZ* expression. Noteworthy, *exoY-lacZ ex planta* expression was not induced by a plant shoot extract (**Figure 1C**) nor nodule extract thus indicating that cAMP-dependent *exo* gene expression and *smc02178* expression are under different genetic control. Instead a plant extract had a negative effect on *exoY* expression that was, however, independent of Clr.

**FIGURE 2:**
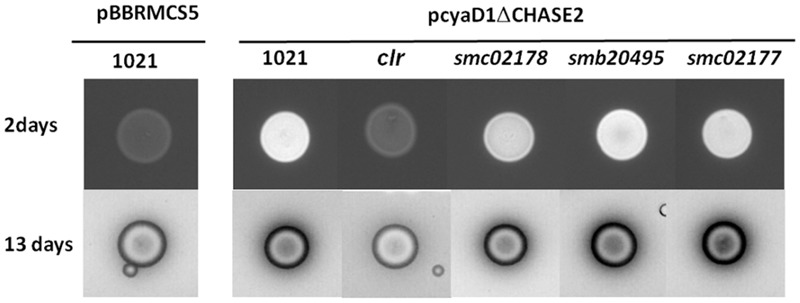
Calcofluor white detection of _endo_cAMP-dependent succinoglycan production in different *S. meliloti* genetic backgrounds after 2 and 13 days incubation of plates at 28°C. The pCyaD1ΔCHASE2 plasmid produces _endo_cAMP constitutively. Pictures at 13 days are inversed images that better show the halo of LMW succinoglycan. The pictures shown have been taken from one of 3 independent replicates. Original images are shown in Supplementary Figure S1.

In order to directly assess the implication of Clr in succinoglycan biosynthesis, we monitored fluorescence of bacterial colonies on agar plates containing the calcofluor white dye which is specific for succinoglycan in strain Rm1021 (Jones, 2012). Under conditions where wt *S. meliloti* showed only background fluorescence, bright fluorescence was triggered (**Figure 2**) in a wt strain carrying the pGMI50127 plasmid that constitutively synthesizes endogenous cAMP (_endo_cAMP) (Tian et al., 2012). Bright fluorescence was almost completely abolished in the *clr* mutant in agreement with previous results (Krol et al., 2016) whereas inactivation of the *smc02178* gene had no detectable effect on overall succinoglycan synthesis (**Figure 2**).

The presence of a halo of similar size corresponding to diffusible low molecular weight succinoglycan around the wt and *smc02178* bacterial colonies indicated that succinoglycan secretion was not impaired in the *smc02178* mutant (**Figure 2**).

### *clr* Negatively Controls Motility

*exo* and *fla* genes show opposite regulation in many biological instances, including symbiosis and stress responses (Mendrygal and Gonzalez, 2000; Ruberg et al., 2003; Hellweg et al., 2009). We confirmed by RT-qPCR that *clr* regulates *flaB* transcription negatively, in accordance with transcriptome experiments (Supplementary Figure S2). A swimming motility assay showed a reduced swim radius of the wild type strain producing _endo_cAMP that was not due a reduced growth of the strain carrying plasmid pGMI50127 (Supplementary Figure S2). Inhibition of motility by _endo_cAMP was indeed mediated by *clr* as observed before (Krol et al., 2016).

### Two Novel Clr-Target Genes Involved in the Control of Secondary Infection

Among the 25 genes of unknown biological function activated by Clr we characterized two of them; *smb20495*, which was the most highly-induced gene in the transcriptome experiment (**Table 1**) and *smc02177*, the gene located next to *smc02178* on the chromosome.

**FIGURE 3:**
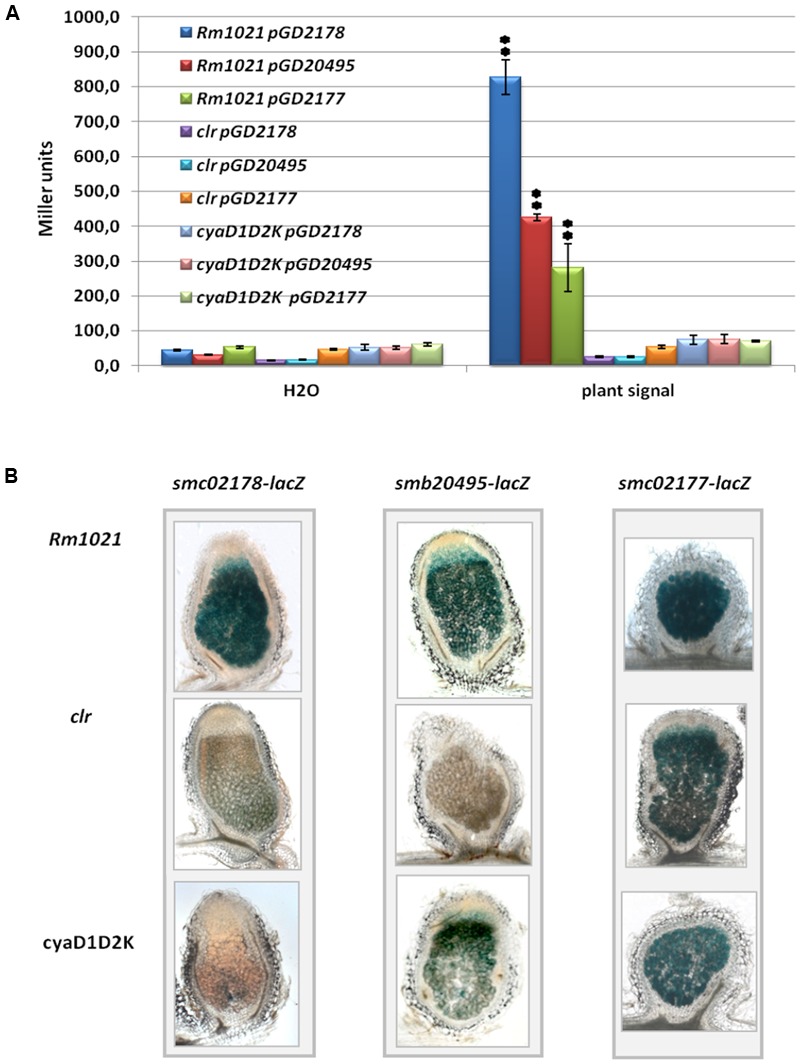
*smc02177* and *smb20495* gene expression. **(A)** Activation of *smc02178-lacZ, smb20495-lacZ* and *smc02177-lacZ fusions* by a *Medicago* shoot extract *ex planta.* ^∗∗^Indicates statistical significance at *p* < 0.01 with respect to water control. **(B)** *smc02178-lacZ, smb20495-lacZ and smc02177-lacZ* expression *in planta*, respectively. The images featuring *smc02178-lacZ* expression have been retrieved from Tian et al. (2012) for comparison.

We first tested activation of *smb20495*- and *smc02177*-*lacZ* translational gene fusions in free-living cultures supplemented by a plant shoot extract known to activate *smc02178* expression (Tian et al., 2012). We found that free-living expression of both genes was activated by the plant signal in a *clr*- and *cyaD1D2K*-dependent manner, although to a lower level as compared to *smc02178* (**Figure 3A**).

*In planta*, the pattern of *smb20495-lacZ* expression in nodules was similar to the one previously described for *smc02178* (**Figure 3B**) and expression strictly depended on *clr*. However, although 30% of the nodules induced by the *cyaD1D2K* mutant were colorless, 70% stained pale blue. Probably one of the many ACs in the *S. meliloti* genome (25 besides CyAD1CyaD2CyaK; Galibert et al., 2001) allows low level of *cyaD1D2K*-independent expression of the *smb20495* gene *in planta*.

The situation was even more contrasted for the *smc02177-lacZ* fusion. Although *smc02177* displayed very little *clr*-independent expression *ex planta* (**Figure 3A**), its expression in mature nodules was essentially *clr* and *cyaD1D2K*-independent (**Figure 3B**). A likely explanation is that a Clr-independent promoter gets strongly activated in nodules.

**FIGURE 4:**
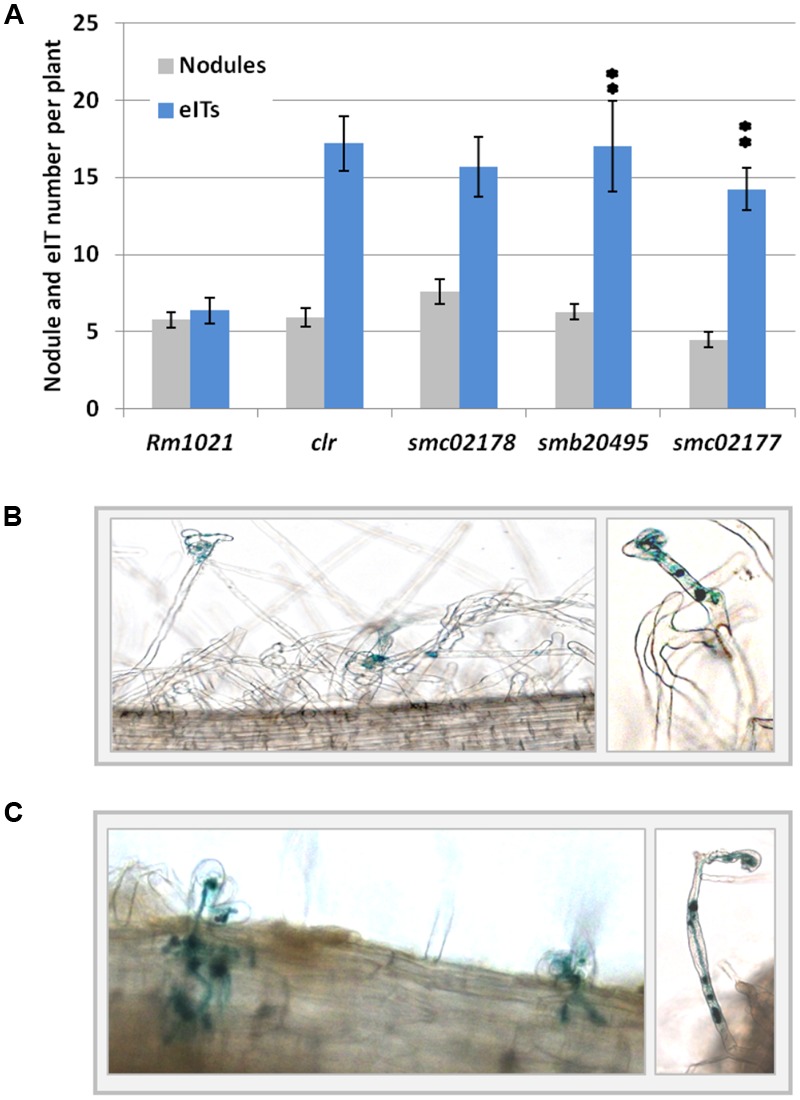
*smb20495* and *smc02177* control secondary infection. **(A)** Nodule and eITs number counting. ^∗∗^Indicates statistical significance at *p* < 0.01 with respect to Rm1021. Data for *clr* and *smc02178* have been retrieved here from Tian et al. (2012) and are shown here for comparison. **(B,C)** *Medicago sativa* roots hyperinfected by *smb20495 and smc*02177 mutants, respectively. Note occasional defense reactions and aborted eITs.

Null mutants of *S. meliloti* in *smb20495* and *smc02177* genes displayed a full hyper-infection phenotype on *M. sativa* (**Figure 4**) comparable to the one previously observed for a *smc02178* or *clr* mutant (Tian et al., 2012). However, no difference in nodule number was observed (**Figure 4A**). Nodules were pink and leaves were green thus indicating that the mutants were not affected for nitrogen fixation, as demonstrated before for a triple *cyaD1D2K* mutant (Tian et al., 2012). Both mutants synthesized _endo_cAMP-dependent HMW and diffusible LMW succinoglycan on calcofluor white dye plates, similar to the *smc02178* mutant (**Figure 2**).

Altogether these results indicate that *smb20495* and *smc02177* expression are prone to plant signal regulation under *clr* and *cyaD1D2K* control. Nevertheless *smc02177* showed a high-level of plant signal-independent symbiotic expression in nodules. Both genes were required for the control of secondary infection.

### Sequence Analysis of the SMc02177, SMc02178 and SMb20495 Proteins

PSORTb 3.0 analysis (Yu et al., 2010) predicted a non-cytoplasmic location for all three SMc02177, SMc02178 and SMb20495 proteins without specifying more precisely their subcellular localization. SignalP 4.1 analysis (Petersen et al., 2011) indicated the presence of a cleavable signal peptide in SMc02178 and SMb20495. As for SMc02178, we experimentally demonstrated the localization of the main portion of the protein in the bacterial periplasm using a combination of *lacZ* and *phoA* reporter fusions (Supplementary Figure S3).

Contrary to SMc02178 for which sequence inspection gave no structural or functional clues, we identified a FecR domain (IPR006860, PF04773) in SMc02177. In the *E. coli* full-length FecR protein, the FecR domain interacts with the amino-terminal periplasmic part of the FecA outer membrane protein involved in iron citrate signaling. Sequence inspection of the large SMb20495 protein indicated the presence of several TPR domains (IPR019734) known to mediate protein–protein interactions.

### The *smc02177, smc02178* and *smb20495* Genes Are Not Required for Plant Signal Perception

**FIGURE 5:**
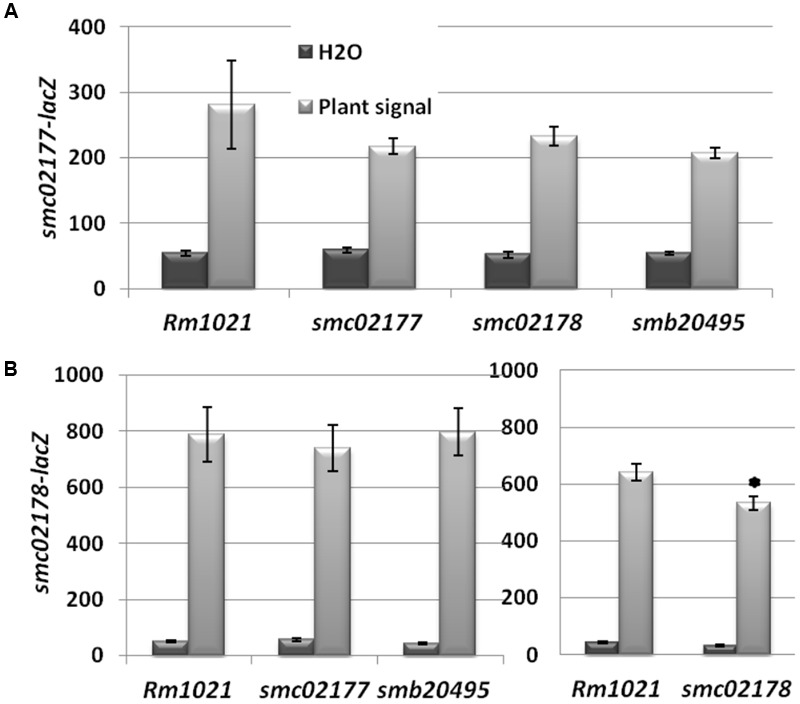
*smc02177, smc02178* and *smb20495* are not involved in plant signal perception. **(A)** *smc02177-lacZ* expression (expressed as Miller Units). **(B)** *smc02178-lacZ* expression. ^∗^Indicates a statistically significant difference at *p* < 0.05 with respect to Rm1021 control.

The predicted extra-cytoplasmic location of the three proteins and the presence of protein–protein interaction motifs in two of them suggested to us the possibility that these proteins were involved in the perception/transduction of the plant signal that activates the CyaD1, CyaD2 and CyaK ACs (Tian et al., 2012). We therefore monitored plant shoot signal-dependent expression of plasmidic *smc02177- and smc02178-lacZ* fusions in the three mutant backgrounds. For the *smc02177-lacZ* fusion no statistically significant difference in expression was observed in any mutant background as compared to wild-type. For the *smc02178* mutation a weak but significant (*p* < 0.05) effect was observed on *smc02178-lacZ* expression (**Figure 5**).

Altogether, none of the genes under test had a significant effect on plant signal perception that could account for its hyper-infection associated phenotype (see Discussion).

## Discussion

### The Clr Regulon

In this work, we compared the transcriptome of a strain moderately overexpressing *clr* (6- to 8-fold, **Table 1**) to that of a *clr* null mutant. As a result we identified 72 genes that showed at least a twofold up- or down-regulation in two biological replicates of the two biological conditions tested, i.e., aerobic and microaerobic (2% O_2_) conditions.

Krol et al. (2016) recently determined the transcriptome of a strain overexpressing the CyaJ AC to a control strain carrying an empty vector plasmid with the purpose of identifying cAMP-dependent genes in *S. meliloti*. Of the 42 genes induced by Clr in both aerobic and microaerobic conditions in our experiments, 27 were also identified as being activated by CyaJ using similar thresholds (*M* > 1 and *p* < 0.05). These 27 genes are thus *bona fide* cAMP-dependent Clr targets. Five additional genes in our experiments were found in the immediate vicinity of these *bona fide* targets. *cyaF2* is a Clr target solely detected in our experiments but for which a Clr-binding site was experimentally identified (Krol et al., 2016). CyaF2 is thus probably a genuine Clr (direct) target too. Altogether, we believe that at least 32 genes (over 42) detected here are *bona fide* Clr- and cAMP-activated targets.

In contrast the list of Clr-repressed genes in our experiments and in previous (Krol et al., 2016) experiments (33 and 82 genes, respectively) showed a very limited overlap; 7 genes all involved in chemotaxis and swimming motility. Metabolic genes evidenced by manipulating intracellular cAMP concentration (Krol et al., 2016) were not detected here and thus may not be Clr targets.

Our data also provide direct evidence that the Clr regulon is wider than the *cyaD1D2K* symbiotic signaling cascade since Clr targets such as *exo* and *fla* genes are not regulated by the plant signal nor its cognate ACs, CyaD1D2K. This is best explained by the fact that they are 28 ACs in the *S. meliloti* genome beside CyaD1D2K (Galibert et al., 2001). Altogether this suggests that Clr is a central transcriptional regulator that integrates different environmental signals *via* different ACs, both in the free-living and symbiotic life of *S. meliloti*.

We initially identified a functional palindromic (TGTTN_8_AACA) Clr-box in the *smc02178* promoter region (Tian et al., 2012). Based on DNA-binding assays, Krol et al. (2016) identified a relaxed consensus (HGTYHCNNNNGRWACA) that we have found in the promoter regions of nine predicted Clr targets [*smb20906–smb20908, smc00864, smc00925, smc01136, smc01210, smc02177, smc03985* (*cyaF2*), *smc04164* and *smc04190*]. Upon studying *smb20495* expression, we have observed that a promoter extending 500 bp upstream of the start ATG codon and carrying the Clr-like box (HGTYHCNNNNGRWACT) was indeed inducible by Clr and cAMP (**Figure 3A**) whereas a shorter promoter version (285 bp upstream of ATG) lacking this putative Clr-box was not (Krol et al., 2016). On this ground we tentatively propose a novel HGTYHCNNNNGRWACW Clr-box consensus that we have also found in the *smb21329* promoter (**Table 1** and Supplementary Table S3). We acknowledge that a functional binding assay is needed to validate this prediction. No functional (e.g., nCRNA) or structural element (e.g., RIME) was identified so far in the 500 nt region between the Clr-box and the *smb20495* start codon^2^.

In summary, 10 to 12 Clr-activated genes identified in this work could be direct Clr targets. Nine of them are chromosomal and three located on pSymB. Noteworthy pSymB-located *exo* genes and the gene cluster encoding the putative polysaccharide (*nodP2-smb21248*) may be indirect Clr targets. Similarly, none of the Clr-repressed genes involved in flagellin synthesis and chemotaxis showed a potential Clr-box matching the [HGTYHCNNNNGRWACW] consensus. So, Clr-repressed genes are likely indirect targets. Although more work is needed to precisely define the full Clr (direct) regulon our data suggest that the Clr-regulon mainly consists of chromosome-located genes positively regulated by Clr.

### Clr-Mediated Control of Legume Root Secondary Infection

We have described here two novel Clr-target genes strictly required for the control of secondary plant infection. Inactivation of any of these two genes led to a hyperinfection phenotype similar to the one observed with a *smc02178, clr* or a triple *cyaD1cyaD2cyaK* mutant. Hence, both genes have a unique and essential role in the control of secondary infection. Similar to *sm02178, smb20495* and *smc02177* were expressed at a very low level *ex planta* in standard culture conditions and were activated by the plant signal *ex planta* in a *cyaD1D2K-* and *clr-*dependent manner. All three genes were expressed in nodules. Yet *smc02177* expression in mature nodules was essentially independent of *clr* and *cyaD1cyaD2cyaK* (**Figure 3B**), likely reflecting a dual regulation of this gene.

The mechanism by which the three Clr-targets restrict secondary infection now needs to be elucidated. No enzymatic function was predicted for any of the corresponding proteins. Instead two of them displayed protein–protein interaction motifs (TPR) or domain (FecR).

We speculated that (some of) these proteins could be involved in plant signal perception. Our present results, however, do not lend support to this possibility (**Figure 5**). However, the observation that the CyaD1, CyaD2 and CyaK ACs act at successive stages of nodule development suggested that different signal molecules could be associated with them (Tian et al., 2012) and indeed we have obtained genetic evidence for other(s) signal in addition to the one evidenced so far in nodules and shoots. Hence the role of the *sm02178, smb20495* and *smc02177* genes in signal sensing would have to be reevaluated if more signals were discovered.

A second possibility is that the *sm02178, smb20495* and *smc02177* genes are involved in the secretion of an effector molecule or MAMP whose recognition by a plant receptor may dampen root susceptibility to infection. In this respect, the transcriptome profiling experiment pointed to two candidate MAMP molecules. The first candidate is cAMP-dependent succinoglycan. Considering the well-established (positive) role of succinoglycan in primary infection it is tempting to speculate that succinoglycan may play a negative role during secondary infection as well. Two lines of evidence, however, argue against implication of cAMP-dependent succinoglycan in the control of secondary infection: (i) the individual *smc02177, smc02178* and *smb20495* mutants -although displaying a full hyperinfection phenotype- were not affected in cAMP-dependent succinoglycan synthesis nor secretion (**Figure 2**) (ii) cAMP-dependent *exoY* gene did not depend on the plant shoot signal (**Figure 1C**) for expression *ex planta*, contrary to genes (*smc02177, smc02178*, and *smb20495*) involved in infection control. Altogether the data suggest that succinoglycan by itself is not the MAPM molecule but does not exclude succinoglycan is part of the MAMP biosynthetic or export pathway.

The second candidate molecule is a polysaccharide compound putatively encoded by a *ca.* Thirty kilobyte *nodP2-smb21248* gene cluster in which we identified at least seven potential Clr target genes by transcriptome profiling. This cluster altogether encompasses 13 glycosyltransferases, 2 sugar epimerases (SMb21228 and SMb21232), 2 tyrosine kinases (SMb21240 and ExoP2) and 3 succinoglycan-related transport proteins (Wzx1, ExoF2, and ExoF3) potentially involved in surface polysaccharide synthesis. Activation by Clr of *nodP2Q2* involved in sulfate activation as well as three sulfotransferases (SMb21237, SMb21243, and SMb21249) in this large cluster as well as the *lpsS-*encoded sulfotransferase (Cronan and Keating, 2004) on the chromosome suggests a sulfated polysaccharide. Both (cAMP-independent) sulfated LPS and KPS have been described in *S. meliloti* (Cronan and Keating, 2004; Townsend et al., 2006). Although highly unlikely, the synthesis of a modified Nod factor molecule cannot be completely excluded at this stage.

The structural characterization of cAMP-dependent polysaccharide molecule and their possible implication in the control of secondary infection is now under investigation. This together with the precise subcellular localization of the SMc02177 and SMb20495 proteins as well as the identification of their interacting partners should shed further light to the control of secondary infection in the *S. meliloti–Medicago* symbiosis.

## Author Contributions

LZ, AG, CMD, FS and AMG performed experiments. AMG, JB and CMB designed the research. CMB, AMG and JB wrote the manuscript.

## Conflict of Interest Statement

The authors declare that the research was conducted in the absence of any commercial or financial relationships that could be construed as a potential conflict of interest.

## Footnotes

1 https://iant.toulouse.inra.fr/bacteria/annotation/cgi/rhime.cgi

2 https://iant.toulouse.inra.fr/bacteria/annotation/cgi/rhime.cgi
