# Supplementary material for: Transcriptomic Insight in the Control of Legume Root Secondary Infection by the Sinorhizobium meliloti Transcriptional Regulator Clr
Source: Front Microbiol. 2017 Jul 6;8:1236. doi: 10.3389/fmicb.2017.01236 (PMC5498481; doi:10.3389/fmicb.2017.01236)
Supplement: Supplementary file 1 [file Table_1.DOCX]

**Table S1:** Bacterial strains and plasmids used in this study

| **Strain or plasmid** | **Description** | **Reference/Source** |
| --- | --- | --- |
| Rm1021 | Str^R^ derivative of *S. meliloti* SU47 | Meade et al. 1982 |
| GMI11566 | 1021 *smc02178*::pVO155, Str^r^, Neo^r^ | Tian et al. 2012 |
| GMI11567 | 1021 *clr*::pVO155, Str^r^, Neo^r^ | Tian et al. 2012 |
| GMI 11896 | 1021 (pFA2175), Str^r^ *,* Gen^r^ | This work |
| GMI12006 | *1021 smb20495::pVO155,* Str^r^, Neo^r^ | This work |
| GMI12007 | *1021 smc02177::pVO155,* Str^r^, Neo^r^ | This work |
| Rm8002 | *phoA* derivative of Rm1021 | Long et al. 1988 |
| DH5α | *E. coli fhuA2 Δ(argF-lacZ)U169 phoA glnV44 Φ80 Δ(lacZ)M15 gyrA96 recA1 relA1 endA1 thi-1 hsdR17* | Bethesda Research Laboratories |
| pBBR1MCS-5 | Cloning vector ; Gen^R^ | Kovack et al. 1994 |
| pGMI50127 (=pcyaD1∆chase2) | pBBR1MCS-5 derivative expressing a truncated *cyaD1* gene; Gen^R^ | Tian et al. 2012 |
| pRK600 | Helper conjugative plasmid, ColE1 replicon with RK2 transfer region; Chl^R^ | Finan et al. 1986 |
| pFAJ1708 | Stable RK2-derivative, constitutive *nptII* promoter, GenR | Dombrecht et al. 2001 |
| pFA2175 | pFAJ1708 expressing the *clr* gene, Genr | This work |
| pVO155 | pUC119 derivative for insertional inactivation in *S. meliloti*, Kan ^r^, Amp ^r^ | Oke and Long 1999 |
| pGD2178 | Beginning of *smc02178* gene fused to *lacZ* cloned into pGD926 (=Ssmc02178-lacZ) | Tian et al. 2012 |
| pGMI50322 | *smc02178* gene fused to *lacZ* cloned into pGD926 (=Lsmc02178-lacZ) | This work |
| pGMI50323 | *smc02178* gene fused to *phoA* cloned into pGD926 (=Ssmc02178-phoA) | This work |
| pGMI50324 | Beginning of *smc02178* gene fused to *phoA* cloned into pGD926 (=Lsmc02178 –phoA) | This work |
| pGD926 | pRK290 derivative containing a promoterless lacZ gene, Tet^R^ | Ditta et al. 1985 |
| pstb-LAFR5-ExoY | pLAFR5 carrying the smb21651 promoter fragment, and *exoY* ORF fragment + ribosome binding site , Tet^R^ | Jones 2012 |
| pGD20495 | pGD926 derivative containing the *smb20495* promoter region fused to *lacZ* | This work |
| pGD2177 | pGD926 derivative containing the *smc02177* promoter region fused to *lacZ* | This work |

**References:**

Ditta, G., Schmidhauser, T., Yakobson, E., Lu, P., Liang, X.W., Finlay, D.R., Guiney, D., and Helinski, D.R. 1985. Plasmids related to the broad host-range vector, pRK290, useful for gene cloning and for monitoring gene expression. Plasmid 13:149-153.

Dombrecht, B., Vanderleyden, J., and Michiels, J. 2001. Stable RK2-derived cloning vectors for the analysis of gene expression and gene function in gram-negative bacteria. Molecular Plant-Microbe Interactions 14:426-430.

Finan, T.M., Kunkel, B., Devos, G.F., and Signer, E.R. 1986. 2^nd^ symbiotic megaplasmid in *Rhizobium meliloti* carrying exopolysaccharide and thiamine synthesis genes. Journal of Bacteriology 167:66-72.

Jones, K.M. 2012. Increased Production of the Exopolysaccharide Succinoglycan Enhances *Sinorhizobium meliloti* 1021 Symbiosis with the Host Plant *Medicago truncatula*. Journal of Bacteriology 194:4322-4331.

Kovach, M.E., Phillips, R.W., Elzer, P.H., Roop, R.M., and Peterson, K.M. 1994. pBBR1MCS – a broad host-range cloning vector. Biotechniques 16:800-802.

Long, S., McCune, S., and Walker, G.C. 1988. Symbiotic loci of *Rhizobium meliloti* identified by random TnPhoA mutagenesis. Journal of Bacteriology 170:4257-4265.

Meade, H.M., Long, S.R., Ruvkun, G.B., Brown, S.E., and Ausubel, F.M. 1982. Physical and genetic characterization of symbiotic and auxotrophic mutants of *Rhizobium meliloti* induced by transposon Tn5 mutagenesis. Journal of Bacteriology 149:114-122.

Oke, V., and Long, S.R. 1999. Bacterial genes induced within the nodule during the Rhizobium-legume symbiosis. Molecular Microbiology 32:837-849.

Tian, C.F., Garnerone, A.M., Mathieu-Demaziere, C., Masson-Boivin, C., and Batut, J. 2012. Plant-activated bacterial receptor adenylate cyclases modulate epidermal infection in the *Sinorhizobium meliloti*-*Medicago* symbiosis. Proceedings of the National Academy of Sciences of the United States of America 109:6751-6756.
