# Supplementary material for: Transcriptomic Insight in the Control of Legume Root Secondary Infection by the Sinorhizobium meliloti Transcriptional Regulator Clr
Source: Front Microbiol. 2017 Jul 6;8:1236. doi: 10.3389/fmicb.2017.01236 (PMC5498481; doi:10.3389/fmicb.2017.01236)
Supplement: Supplementary file 2 [file Table_2.DOCX]

**Table S2.** Primers used in this study.

| **Table S**   \| **Gene** \| **Primer name** \| \| **Sequence 5′ to 3′** \| \| --- \| --- \| --- \| --- \| \| RplM \| RplM L \| \| CAGAGGTGGAGAAGAAGTGG \| \| RplM \| RplM R \| \| GCCACCTACACGCCGCATGT \| \| ExoH \| ExoH L \| \| GCTCCTTCTTTGTGGTCGTC \| \| ExoH \| ExoH R \| \| GAAATACAGGGGCAGGTTGA \| \| ExoY \| ExoY L \| \| AACGCAGTTTCGACGTTCTT \| \| ExoY \| ExoY R \| \| CTTGAAGGATTGGCCATTGT \| \| ExoM \| ExoM L \| \| GTCTGGGCAAAAGGTGAGAT \| \| ExoM \| ExoM R \| \| GGTGAAGAAATCCGTGTCCTC \| \| ExoN \| ExoN L \| \| AGTGCCCAAGGAGATGTTGAC \| \| ExoN \| ExoNR \| \| GGCTGGTGACGAAGACGATA \| \| FlaB \| FlaB-669U \| \| GACCTTCGACGGCGACTATG \| \| FlaB \| FlaB-786L \| \| GACGACTTCCTGACCCGTTG \| \| Smc02178 \| 2178-BamHILacZ \| \| CGGGATCCTGCAATCCGTCCGTGGTCGCCTGCG \| \| phoA \| phoA-EcoRV \| \| GATATCCCTGTTCTGGAAAACCGGGCTGCTCAGGGC \| \| phoA \| phoA-BamHI \| \| CGGGATCCGGGACATGTTTATTTCAGCCCCAGAGC \| \| Smc02178 \| 2178-EcoRVphoAc \| \| GATATCCAGGCTCGATCCAACGTGTTTCTTCAT \| \| Smc02178 \| 2178-EcoRVphoAL \| \| GATATCCTGCAATCCGTCCGTGGTCGCCTGCG \| \| Smc02178 \| 2178H \| \| CGAAGCTTCGTCAGGACATAATCCTTGTCGAG \| \| Smc02178 \| L2178 \| \| CTCAACAGGGCTGGACAGA \| \| Smc02178 \| R2178 \| \| CAAGGAGATCGGCATAGCTG \| \| clr \| REco2175 \| \| CGGAATTCGCTTTTGGGCAAGCGGC \| \| clr \| LBamH2175 \| \| GCGGATCCGCGTAAAGGGGAACGCA \| \| Smb20495 \| 20495 L \| \| GGATCCAACAGTTCAGCGGCAACC \| \| Smb20495 \| 20495R \| \| TCTAGA TGTACGTTCGGTGTCTGGAG \| \| Smb20495 \| SMb20495 HindIII \| CCAAGCTT GAG GAA AGG CCA ACG GTC TCC CG \| \| \| Smb20495 \| SMb20495 BamHI \| CGGGATC CGC TAC GGA CAG AGC GCT GGA GAG \| \| \| Smc02177 \| L02177 \| GGATCCGCGGTTCTCGACAAGGATTA \| \| \| Smc02177 \| R02177 \| TCTAGAGACACCACGCCATGTTCC \| \| \| Smc02177 \| p2177HindIII \| CCCAAGCTTGAGGCAAGACCACGGCG \| \| \| Smc02177 \| p2177 bamH1 \| CGCGGATCCCG TCT CCT CAT CTC GAA CTG \| \| |  |
| --- | --- | --- | --- | --- | --- | --- | --- | --- | --- | --- | --- | --- | --- | --- | --- | --- | --- | --- | --- | --- | --- | --- | --- | --- | --- | --- | --- | --- | --- | --- | --- | --- | --- | --- | --- | --- | --- | --- | --- | --- | --- | --- | --- | --- | --- | --- | --- | --- | --- | --- | --- | --- | --- | --- | --- | --- | --- | --- | --- | --- | --- | --- | --- | --- | --- | --- | --- | --- | --- | --- | --- | --- | --- | --- | --- | --- | --- | --- | --- | --- | --- | --- | --- | --- | --- | --- | --- | --- | --- | --- | --- | --- | --- | --- | --- | --- | --- | --- | --- | --- | --- | --- | --- | --- | --- | --- | --- | --- | --- | --- | --- | --- | --- | --- | --- | --- | --- | --- | --- | --- | --- | --- | --- | --- | --- |
