# Supplementary material for: Transcriptomic Insight in the Control of Legume Root Secondary Infection by the Sinorhizobium meliloti Transcriptional Regulator Clr
Source: Front Microbiol. 2017 Jul 6;8:1236. doi: 10.3389/fmicb.2017.01236 (PMC5498481; doi:10.3389/fmicb.2017.01236)
Supplement: Supplementary file 3 [file Table_3.DOCX]

**Table S3 :** Clr-boxes upstream of Clr-target genes (see text for details)

| **Gene ID** | **Position*** | **Clr- box** |
| --- | --- | --- |
| *smb20495* | 499 | TGTT ACTTCCGG AACT |
| *smc04190* | 88 | TGTC ACATCCGG AACA |
| *cyaF2* | 86 | CGTT CCCCGAGA AACA |
| *smc02178* | 52 | TGTT TCCCGCGG AACA |
| *smb20906* | 117 | AGTT CCGCGAGG AACA |
| *smc04164* | 157 | TGTT ACCCGCGG AACA |
| *smc00864* | 430 | TGTT CCGTTTGG AACA |
| *smc01210* | 637 | TGTT CCCCGGGA AACA |
| *smc02177* | 132 | TGTT CCGCGGGA AACA |
| *smb21329* | 71 | TGTT ACCCAGGG AACT |
| *smc01136* | 291 | AGTT TCCCACGG AACA |
| *smc00925* | 101 | TGTT CCCTCAGA TACA |
| **Consensus** |  | **HGTY HCNNNNGR WACW** |

*distance between ATG and the first nucleotide position of the Clr box
