# Supplementary figures and images for: Transcriptomic Insight in the Control of Legume Root Secondary Infection by the Sinorhizobium meliloti Transcriptional Regulator Clr
Source: Front Microbiol. 2017 Jul 6;8:1236. doi: 10.3389/fmicb.2017.01236 (PMC5498481; doi:10.3389/fmicb.2017.01236)

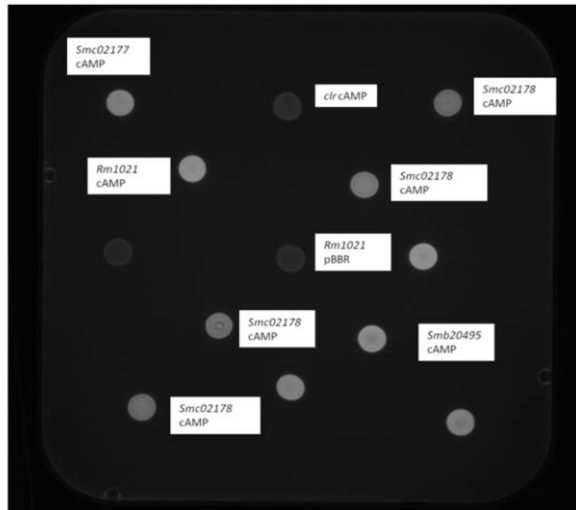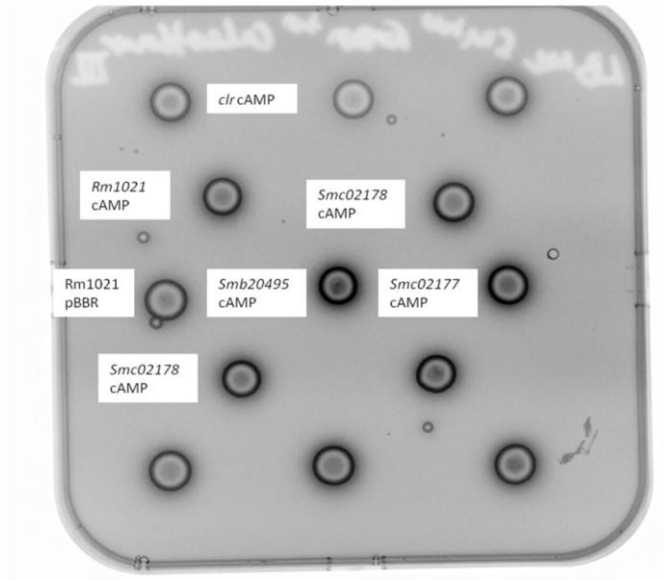

Figure S1: Original calcofluor white dye images used for generating Figure 2.

Supplement: Supplementary file 4 [file Image_1.PDF]
