# Supplementary material for: Transcriptomic Insight in the Control of Legume Root Secondary Infection by the Sinorhizobium meliloti Transcriptional Regulator Clr
Source: Front Microbiol. 2017 Jul 6;8:1236. doi: 10.3389/fmicb.2017.01236 (PMC5498481; doi:10.3389/fmicb.2017.01236)
Supplement: Supplementary file 5 [file Image_2.PDF]

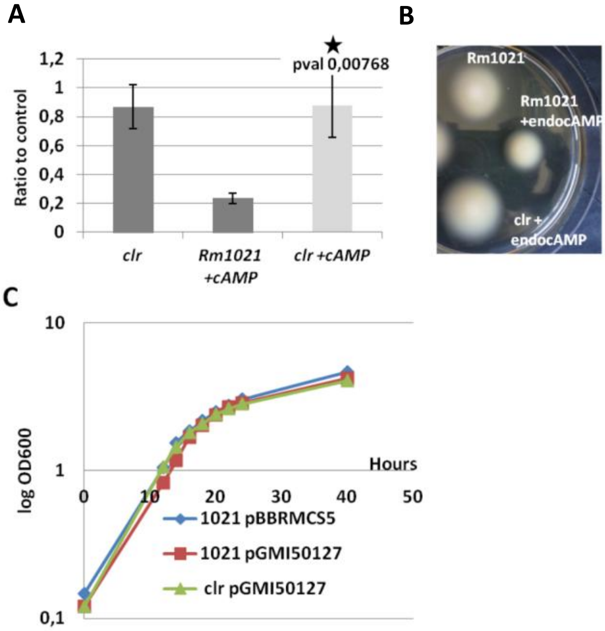

Figure S2: *Clr* represses motility. Panel A: RT-qPCR analysis of *flaB* gene expression in the presence of $_{exo}$cAMP (5mM) expressed as a ratio to *flaB* expression in wt Rm 1021 strain with no cAMP added. Panel B: Bacterial swimming motility assay on LBMC 0.2% agar plates. $_{endo}$cAMP was produced by plasmid pGMI50127 Panel C: Endogenous cAMP production by pGMI50127 plasmid does not impair bacterial growth. Growth was in Vincent minimal medium supplemented with mannitol (1%w/vol) and glutamate (0.1%) at 28°C.”
