# Supplementary material for: Transcriptomic Insight in the Control of Legume Root Secondary Infection by the Sinorhizobium meliloti Transcriptional Regulator Clr
Source: Front Microbiol. 2017 Jul 6;8:1236. doi: 10.3389/fmicb.2017.01236 (PMC5498481; doi:10.3389/fmicb.2017.01236)
Supplement: Supplementary file 6 [file Image_3.PDF]

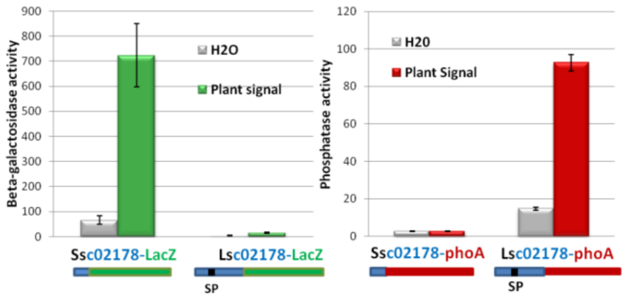

Figure S3: Localization of the Smc02178 protein in the periplasm. Use of the *phoA* fusion showed that the main portion of the Smc02178 protein is in the periplasm. Activity required the presence of a signal peptide (SP) that has a very well predicted cleavage site (SignalP) between positions 31 and 32.
